# Exposing tobacco companies’ retail presence and highlighting regulatory options

**DOI:** 10.18332/tpc/211478

**Published:** 2026-04-11

**Authors:** Hussein A. Faeq, Rosemary Hiscock, Raouf Alebshehy

**Affiliations:** 1 Tobacco Control Research Group, Centre for 21st Century Public Health, Department for Health, University of Bath, Bath, United Kingdom

**Keywords:** policy solutions to tobacco retailing, targeted tobacco marketing, retailer incentive programs, tobacco industry targeting retail environment

## Abstract

Although much is already known about the strategies that tobacco companies use to generate profits (and consequently compromise health), and options for retail regulation, industry tactics within the retail environment have not been comprehensively collated. Tobacco companies’ billions of dollars of investments in retail play a vital role in normalizing tobacco products in everyday life because they are an everyday sight. These investments include for example retailer incentives, instore advertising and display stands. Companies continue to promote their tobacco products indirectly through retailer-related activities in many countries because they are often not included in governments’ tobacco marketing restrictions. In light of this, in this monitoring letter, we highlight some of the industry's tactics to control the retail environment and discuss potential solutions to counter its strategic presence in retail.

## INTRODUCTION

### Retailer incentive schemes

Although restriction of tobacco companies’ retailer incentive program is included in treaty obligations of Article 13 of the World Health Organization Framework Convention on Tobacco Control (WHO FCTC) which covers tobacco advertising, promotion, and sponsorship (TAPS), unfortunately, only a limited number of countries have barred these programs^1-3^.

Financial agreements secured between the transnational tobacco companies’ (TTCs’) representatives and retailers are often privately negotiated and usually contain clauses granting industry representatives control over the placement of tobacco products in retail settings^2,4^. These contracts may also be signed by retailers without fully understanding the terms after receiving some oral explanation^5,6^. Studies indicate participation in such schemes can put retailers under implicit pressure to meet companies’ expectations^2,7,8^.

Incentives to encourage retailers to maintain the optimal placement of TTCs’ products on store shelves can include financial benefits such as direct payments, discounted stock, and reward programs^9,2,10,11^. In countries where the display of tobacco products is banned, industry representatives encourage retailers to verbally promote their own products in return for some cash and bonuses^2,4^. To further strengthen retailers’ loyalty and increase engagement, TTCs also offer experiential incentives beyond financial rewards, such as sponsored events, luxury travel and exclusive rewards^12,13^.

Reports from former tobacco industry employees and retailers in Australia confirm two things: 1) tobacco companies allocate significant budgets to retailer incentives; and 2) these budgets are used to maintain product visibility by taking advantage of regulatory loopholes in existing tobacco control laws^14,11,12^. Such practices, however, undermine the comprehensive marketing bans intended by the WHO FCTC treaty^11^.

## COMMENTARY

### Targeted marketing to specific demographics

Tobacco companies carefully employ in-store marketing techniques to target demographic groups based on ethnicity, gender, and age^2^. Tobacco retailers are present at greatest density within areas with high levels of socioeconomic disadvantage and a high ethnic diversity; they sell tobacco products in these areas at low prices to draw more consumers and improve sales^2,15^. In the United States, menthol cigarette advertisements are significantly more prevalent within retail stores in areas with higher proportions of Black and Hispanic residents or near schools with more students^16^.

In recent decades, tobacco retail marketing campaigns have also expanded widely across low- and middle-income countries (LMICs) where the young population, growing economies, and weak regulatory enforcement offer profitable opportunities for the industry to attract young consumers^17-19^. A common practice used by tobacco companies is to encourage retailers to strategically place tobacco products and eye-catching advertising stands at children’s eye level at the point of sale^20^.

In New Zealand, Canada, Australia, and the US, the tobacco industry also specifically targets the indigenous populations through retail strategies. These strategies include promotional allowances in the form of giveaways and coupons directed at retailers and wholesalers, as well as price reductions offered to customers who purchase multiple cartons^15,21,22^. For instance, within some US indigenous territories, tobacco retailers follow the Federal regulations but are not subject to state-level tobacco control policies^22^. Consequently, tobacco retail licensing, TAPS, and other tobacco control policies do not apply to retailers operating in these areas^22,23^. The industry often exploits these regulatory exemptions, specifically tailored to these populations, to implement targeted marketing campaigns^22,23^.

Tobacco companies differentiate marketing to men and women. According to the industry documents, it used point-of-sale marketing tactics, such as in-store discounts affixed to cigarette packs and cents-off pack coupons to specifically target low-income African American women^24,25^. Male smokers have also been targeted with price-reducing promotions such as ‘buy three, get three free discount’ offers on specific brands that are attractive to young men^26^.

### Retailer mobilization against regulations

TTCs frequently argue that tobacco control policy changes could impact retailers’ sales and they often collaborate with retailers to delay, obstruct, and circumvent changes^27^. TTCs and retailers claim that imposing tobacco control policies on retail stores can have disastrous economic consequences, such as the financial burdens associated with adherence to new regulatory mandates (compliance costs), lost revenue, and increased illicit tobacco trade^28-32^. For example, British American Tobacco claimed that proposed licensing requirements in Kenya would push small businesses out of work and disrupt the tobacco market^33^. However, policies can be designed to support retailer transition away from tobacco sales and be consistent with the WHO FCTC treaty. For instance, San Francisco’s ordinance provided incentives supporting corner stores to shift from tobacco to selling fresh fruits and vegetables. This program successfully reached an agreement with the retail association and led to a policy solution that was supported by all stakeholders^9^.

Retailer associations, frequently cited as receiving support from TTCs, have often attempted to delay or block regulations by engaging retailers in legal challenges and lobbying campaigns targeting policymakers^29^. The United Kingdom (UK) Association of Convenience Stores, associated with tobacco industry support, claimed that the display ban would generate significant costs for shopkeepers of up to £10000^30^. However, the UK Government Impact Assessment did not support this claim, and instead estimated a one-off compliance cost ranging from £450 to £850, depending on the size of the shop^30^.

### WHO FCTC and beyond-policy pathways for retail regulation

Measures such as licensing requirements (Article 15 of the FCTC treaty), banning tobacco sales via vending machines, sales to minors (Article 16), and TAPS restrictions (Article 13) all fall under WHO FCTC policies designed to limit tobacco availability and regulate the retail environment. There are also further effective measures that are not directly included in the WHO FCTC^9^. These identified policies can be grouped into main categories: 1) limiting the number of tobacco retailers through restricting their geographical placement via setting minimum distance either between retailers or from specific facilities, or limiting outlet density; 2) restricting sales by retailer type: permit sales only via government-controlled outlets or alternatively ban sales for example from supermarkets; 3) restricting methods of tobacco sales by implementing bans on home delivery and tray or mobile tobacco sales; and 4) reducing accessibility to tobacco products in retail environment by capping purchase amounts, restricting sales hours or days, and minimizing their in-store visibility and proximity^34,9^. Public backing for country-level (non-WHO FCTC) policies relevant to the tobacco retail environment such as restricting tobacco sales around places of residence, youth-oriented facilities, and other sensitive locations exists^35^, although evidence from a variety of countries would be helpful.

## CONCLUSION

Simulations of implementation of tobacco retail regulatory measures suggest they could reduce tobacco availability to youth from 4% to 100%^36^. Such measures, situated within the WHO FCTC framework, could collectively contribute to diminishing tobacco presence in the retail environment and provide policymakers with strategies to counter the tobacco industry’s strong retail presence^36^. These suggested interventions were often developed in specific country contexts. Future research should explore their feasibility and adaptability across different settings, potentially strengthening their applicability to a broader scale.

## Data Availability

Data sharing is not applicable to this article as no new data were created.
